# Emerging Pattern of Rabies Deaths and Increased Viral Infectivity

**DOI:** 10.3201/eid0902.020083

**Published:** 2003-02

**Authors:** Sharon L. Messenger, Jean S. Smith, Lillian A. Orciari, Pamela A. Yager, Charles E. Rupprecht

**Affiliations:** * Centers for Disease Control and Prevention, Atlanta, Georgia, USA

**Keywords:** rabies, cryptic deaths, terrestrial mammals, increased infectivity, Silver-haired Bat, Eastern Pipistrelle, research

## Abstract

Most human rabies deaths in the United States can be attributed to unrecognized exposures to rabies viruses associated with bats, particularly those associated with two infrequently encountered bat species (*Lasionycteris* *noctivagans* and *Pipistrellus* *subflavus*). These human rabies cases tend to cluster in the southeastern and northwestern United States. In these regions, most rabies deaths associated with bats in nonhuman terrestrial mammals are also associated with virus variants specific to these two bat species rather than more common bat species; outside of these regions, more common bat rabies viruses contribute to most transmissions. The preponderance of rabies deaths connected with the two uncommon *L.* *noctivagans* and *P. subflavus* bat rabies viruses is best explained by their evolution of increased viral infectivity.

Bites by rabid dogs are the source of 35,000–50,000 human rabies deaths each year globally (*1*), whereas most human rabies deaths in the United States are attributed to unrecognized exposures to rabid bats. Particular attention has focused upon two relatively rare bat species (*Lasionycteris noctivagans* and *Pipistrellus subflavus*) because rabies variants associated with these species (variants) account for approximately 70% of human cases and 75% of cryptic rabies deaths (*2*–*6*).

Molecular typing (i.e., phylogenetic analysis of DNA data) has shown that rabies viruses associated with insectivorous bats (*L. noctivagans* and *P. subflavus* variants in particular) are the culprits in what otherwise would have been unsolved cryptic human rabies deaths. However, phylogenetic analyses of human rabies cases have not provided insights into why an unexpectedly large proportion of human rabies deaths involve the uncommon *L. noctivagans* and *P. subflavus* variants. Passive surveillance systems used by state public health departments confirm that human encounters with Eastern Pipistrelle bats (*P. subflavus)* and Silver-haired Bats (*L. noctivagans)* are rare. Neither species exceeded 5% of all bats submitted for rabies testing in the southeastern United States, and Silver-haired Bats account for <12% of all bats submitted in the Northwest (*7*). Moreover, the prevalence of rabid individual bats within each species is also low and similar to the estimated prevalence in other, more common, bat species (*8*,*9*). While these surveillance studies are known to produce biased estimates of the true prevalence of rabies in natural bat populations, they should accurately reflect the prevalence of rabies in bat species encountered by the public. In addition, we have determined that although other bat species occasionally are infected with *L. noctivagans* and *P. subflavus* variants, the frequency of such spillover is low (*6*,*10*). A survey of rabid “house bats” (i.e., *Eptesicus* *fuscus* and *Myotis* *lucifugus*) in the United States revealed that only 2 of 117 *E*. *fuscus* and 4 of 15 *M.* *lucifugus* were infected with *L. noctivagans* and *P. subflavus* variants (*6*). The sample size of *M*. *lucifugus* is notably small because this species is rarely found rabid, despite submissions of thousands of individual bats each year (*11*). Thus, all available data suggest that *L. noctivagans* and *P. subflavus* variants are rare.

Given that *L. noctivagans* and *P. subflavus* variants appear to be infrequently encountered, two explanations have been proposed to explain their prevalence among cryptic human rabies deaths associated with bats. The small vector hypothesis suggests a failure to recognize that a bite has occurred when a small bat is involved (*7*,*12*). Absence of a bite history may result from inaccurate documentation when a patient could not be questioned directly or was not lucid. In 18 of 34 cases with no bite history, however, documented contact with a potentially rabid animal could have contributed to an unrecognized bite (i.e., physical contact with bats in 11 cases, bats in residence in 4 cases, and contact with a known sick domestic animal in 3 cases). Among these contacts, 11 involved *L. noctivagans* and *P. subflavus* variants. In addition, bites by smaller bat species, such as Eastern Pipistrelles, may be more likely to go unnoticed than bites by larger bats, which explains why more cases are associated with this species. Wounds from the teeth of small bat species are not easily seen without careful examination (*13*,*14*), possibly leading to the impression that a bite has not occurred.

A second hypothesis was suggested by results from experimental data comparing rabies virus isolates from Silver-haired Bats with those from domestic dogs and a coyote (*15*,*16*). These data showed that, although both viruses replicated equally well in neuroblastoma cells, rabies viruses from Silver-haired Bats replicated to higher titers in fibroblast and epithelial cells, particularly at a temperature of <34°C. Such growth characteristics might facilitate successful infection after a superficial bite. Thus, superficial contact may occur frequently between bats and terrestrial mammals (including humans), but may be unlikely to result in a productive infection unless *L. noctivagans* and *P. subflavus* variants are involved (the increased infectivity hypothesis). Although these experimental data suggest that *L. noctivagans* and *P. subflavus* variants have evolved increased infectivity, comparison between viruses associated with Silver-haired Bats and dogs (including one coyote) is not sufficient to pinpoint whether increased infectivity evolved in the common ancestor of *L. noctivagans* and *P. subflavus* variants and, thereby, is relevant to the noted prevalence of these variants among human rabies deaths.

We proposed a novel test of the increased infectivity hypothesis using a comparative phylogenetic approach in which we characterized transmission patterns from bats to nonhuman terrestrial mammals. Transmission of bat rabies to terrestrial mammals can be viewed as a natural experiment that removes the confounding effects of vector size and, therefore, can be used as a control in understanding transmission patterns between bats and humans. That is, such comparisons take advantage of a fundamental difference between rabies exposures in humans and rabies exposures in other terrestrial mammals (nonhuman terrestrial mammals cannot initiate postexposure prophylaxis even if they are aware of a bite). Given that size of the vector species is not a factor in terrestrial mammal deaths, we can control for the effect of bat vector size in our comparison. Therefore, a disproportionate number of *L. noctivagans* and *P. subflavus* cases among terrestrial mammals would be consistent with increased infectivity of the *L. noctivagans* and *P. subflavus* variants. If these variants are not overrepresented in terrestrial mammal rabies cases, we can reject the increased infectivity hypothesis, leaving the small vector hypothesis as the most plausible alternative.

## Methods

### Sequence Data

Sequences were obtained from reverse transcriptase polymerase chain reaction (RT-PCR)–amplified portions of the nucleoprotein (positions 1,157–1,477), glycoprotein (positions 4,297–4,817), and psi (positions 5,069–5,424) regions based on the reference strain M13215 (*17*). The taxa include 32 rabies virus samples obtained between 1958 and 2000 from frozen (n=27) and formalin-fixed (n=5) U.S. human brain tissue specimens, 17 of 41 U.S. bat species (n=46), and 98 nonhuman terrestrial mammals (24 cats, 3 dogs, 5 cattle, 9 horses, 1 sheep, 1 llama, 42 foxes [grey, red, and kit], 1 raccoon, 1 ringtail cat, and 11 striped skunks), determined to have been infected by a bat rabies virus by using monoclonal antibody screening. Reservoir species from the eight U.S. terrestrial rabies-endemic regions (n=23) were included as outgroups. Nucleotide sequences included in this study are available in GenBank.

### Phylogenetic Analyses

Phylogenetic analyses used PAUP* 4.0b2 (*18*), employing the neighbor-joining search algorithm (minimum evolution) using maximum likelihood to estimate Ti:Tv ratio and nucleotide base frequencies (settings correspond to Hasegawa-Kishino-Yano [HKY] 1985 model of nucleotide sequence evolution). We assessed tree support using the nonparametric bootstrap method (1,000 replicates). Phylogenetic analyses presented here included only frozen human brain samples (n=27). Additional analyses (not shown) that included incomplete taxa (formalin-fixed human case samples) did not alter tree topology but decreased nonparametric bootstrap proportions for nodes, including formalin-fixed taxa.

### Results and Discussion

**Figure 1:**
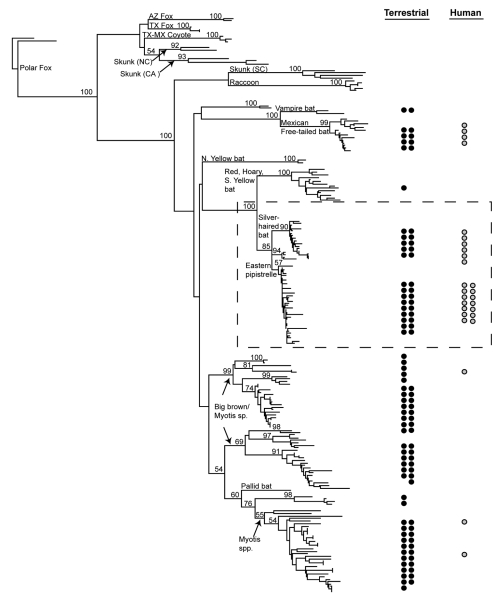
Phylogenetic tree of bat-associated rabies cases. Taxa represent 208 rabies virus variants from 27 human rabies cases (formalin-fixed taxa removed) and 98 terrestrial mammals infected with a bat rabies virus, 60 bat samples representing 17 species, and 23 terrestrial mammal outgroup taxa. Each circle represents a case (terrestrial mammal = closed circles, human = open circles) associated with the monophyletic clade in the phylogeny to the left. Numbers at tree nodes indicate nonparametric bootstrap proportions.

**Figure 2:**
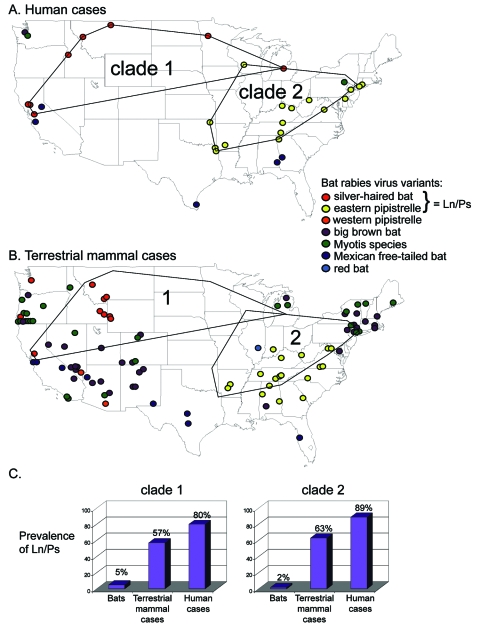
Geographic distribution of (A) human (including 5 formalin-fixed samples not shown in Figure 1) and (B) terrestrial mammal cases identified by rabies virus variant isolated. Minimum length polygons delimiting human cases associated with Silver-haired Bats (clade 1) and Eastern Pipistrelles (clade 2) shown in (A) and superimposed in (B). (C) Prevalence in regions delimited by clades 1 and 2. *Lasionycteris* *noctivagans* (Ln) and *Pipistrellus* *subflavus* (Ps) prevalence in bats was estimated from unpublished state public health department reports that determined the percentage of rabies-positive Silver-haired or Eastern Pipistrelle bats from the total number of bats submitted (7, unpub. data). *L.* *noctivagans* and *P.* *subflavus* prevalence in terrestrial mammals and humans is estimated as percentage of all spillover cases in each clade region infected with *L.* *noctivagans* or *P.* *subflavus*.

Phylogenetic analysis (Figure 1) links 20 of 27 bat-associated human cases (24/32 cases, including formalin-fixed samples not shown in Figure 1) with *L. noctivagans* and *P. subflavus* variants. In contrast to the patterns seen in humans (Figures 1 and 2A), a wide variety of bat rabies variants are implicated in the terrestrial mammal cases across a broad geographic area and largely correspond to the most common bat rabies virus variants in that geographic region (Figures 1 and 2B). Two notable exceptions to this general geographic pattern occur in the northwestern and the southeastern United States (Figure 2B). In these two regions, *L. noctivagans* and *P. subflavus* variants account for a substantially larger percentage of transmission events to terrestrial mammals than expected, given the rarity of the host bat species and *L. noctivagans* and *P. subflavus* variants in those geographic areas (Figure 2C) (because spillover of these variants to other bat species is rare, we used the prevalence of rabid Silver-haired Bats and Eastern Pipistrelles as a surrogate for the prevalence of *L. noctivagans* and *P. subflavus* variants). These two geographic regions also match very closely the geographic distribution of human rabies deaths in the United States associated with these variants (Figure 2A). In the northwest region, delimited by the human cases associated with Silver-haired Bats (clade 1), the *L. noctivagans* rabies virus variant accounted for 57% of bat-associated cases in terrestrial mammals and 80% of bat-associated cases in humans, despite the fact that rabies-positive Silver-haired Bats account for only 5% of all bats submitted for testing. Similarly, in the southeast region, delimited by human cases associated with Eastern Pipistrelles (clade 2), where neither Eastern Pipistrelles nor Silver-haired Bats exceed an average of 2% of all rabies-positive bats submitted, the *P. subflavus* variant accounted for 63% of bat-associated cases in terrestrial mammals and 89% of bat-associated cases in humans. The high prevalence of *L. noctivagans* and *P. subflavus* variants among terrestrial mammals in the same regions where human cases have occurred suggests that a similar mechanism, such as increased infectivity of these rabies virus variants, is responsible for both epidemiologic patterns.

Our study included 13 terrestrial mammal species, both domesticated and wild, suggesting that exposure to bat species likely occurred in a variety of habitats, including more remote forest habitats. Our data set was comprised mostly of foxes and cats (two species likely to capture and feed upon bats), but we had no reason to suspect that their behavior would create a disproportionate opportunity for infection by *L. noctivagans* and *P. subflavus* variants compared with other bat variants.

While our data do not invalidate the small vector hypothesis, the terrestrial mammal data show that even when vector size does not play a role in determining whether bat bites are recognized, *L. noctivagans* and *P. subflavus* variants are still the most prevalent rabies virus variants among bat-associated terrestrial mammal deaths in the northwestern and southeastern United States. For human rabies cases, small vector size may still play a role in the probability of detecting a bat bite, while the increased infectivity of *L. noctivagans* and *P. subflavus* variants enhances the likelihood of a successful infection following a superficial bite. Additional experimental data (e.g., site-directed mutagenesis) will be necessary to show definitively whether *L. noctivagans* and *P. subflavus* variants have evolved genetic changes associated with increased infectivity. Nonetheless, comparisons of phylogenetic patterns between independent datasets in which one data set can be used to control for one or more potentially relevant parameters offers a valid method of hypothesis testing. Additionally, through phylogenetic analyses such as these, we can target those taxa and genomic regions that warrant further investigation.
